# Decreased circulating T regulatory lymphocytes in obese patients undergoing bariatric surgery

**DOI:** 10.1371/journal.pone.0197178

**Published:** 2018-05-14

**Authors:** Claudia Agabiti-Rosei, Valentina Trapletti, Silvia Piantoni, Paolo Airò, Angela Tincani, Carolina De Ciuceis, Claudia Rossini, Francesco Mittempergher, Amin Titi, Nazario Portolani, Stefano Caletti, Maria Antonietta Coschignano, Enzo Porteri, Guido A. M. Tiberio, Paola Pileri, Leonardo Solaini, Rajesh Kumar, Silvia Ministrini, Enrico Agabiti Rosei, Damiano Rizzoni

**Affiliations:** 1 Clinica Medica, Department of Clinical and Experimental Sciences, University of Brescia, Brescia, Italy; 2 Chair of Rheumatology, Department of Clinical and Experimental Sciences, University of Brescia, Brescia, Italy; 3 Clinica Chirurgica, Department of Clinical and Experimental Sciences, University of Brescia, Brescia, Italy; 4 Istituto Clinico Città di Brescia, Division of Medicine, Brescia, Italy; Nagoya University, JAPAN

## Abstract

**Objective:**

It has been previously demonstrated that T lymphocytes may be involved in the development of hypertension and microvascular remodeling, and that circulating T effector lymphocytes may be increased in hypertension. In particular, Th1 and Th 17 lymphocytes may contribute to the progression of hypertension and microvascular damage while T-regulatory (Treg) lymphocytes seem to be protective in this regard. However, no data is available about patients with severe obesity, in which pronounced microvascular alterations were observed.

**Design and methods:**

We have investigated 32 severely obese patients undergoing bariatric surgery, as well as 24 normotensive lean subjects and 12 hypertensive lean subjects undergoing an elective surgical intervention. A peripheral blood sample was obtained before surgery for assessment of CD4+ T lymphocyte subpopulations. Lymphocyte phenotype was evaluated by flow cytometry in order to assess T-effector and Treg lymphocytes.

**Results:**

A marked reduction of several Treg subpopulations was observed in obese patients compared with controls, together with an increased in CD4+ effector memory T-effector cells.

**Conclusion:**

In severely obese patients, Treg lymphocytes are clearly reduced and CD4+ effector memory cells are increased. It may be hypothesized that they might contribute to the development of marked microvascular alterations previously observed in these patients.

## Introduction

The increasing incidence and prevalence of obesity among nearly all countries around the world has a dramatic impact on cardiovascular risk [1,2]. An increased systemic oxidative stress/inflammation is a common accompaniment of obesity [3–6], as suggested by the observation, in several studies, of increased circulating markers, including interleukin-6, C-reactive protein, tumoral necrosis factor alpha and plasminogen activator inhibitor-1 [3–6]. Obese patients, independently from the presence of hypertension, show the presence of microvascular structural alterations, together with endothelial dysfunction [7]. After surgical correction of obesity and consistent weight loss, a significant improvement of microvascular structure and of some oxidative stress/inflammation markers was observed [7].

Recently, it was proposed that both the innate and the adaptive immunity, in particular T effector lymphocytes and T regulatory—Treg–lymphocytes, might be involved in the development of hypertension as well as of cardiovascular diseases in general [8–10]. Mice lacking T and B cells (RAG-1-/- mice) compared with control C57BL/6 do not develop neither hypertension nor abnormalities of vascular function or structure during angiotensin-II infusion or administration of desoxycorticosterone acetate and salt [11]. Adoptive transfer of T, but not of B cells prevented the development of these abnormalities [11]. In animal models, Tregs injection was able to prevent angiotensin II–induced hypertension, as well as vascular injury/inflammation [12]. Data in human hypertension are scarce, however, an increase in circulating interleukin-17A producing CD4+ T cells and both CD4+ and CD8+ T cells that produce interferon-γ were observed in hypertensive patients compared with normotensive controls [13]. In addition, inverse correlations were observed between indices of microvascular structure (in subcutaneous small arteries or in retinal arterioles) and circulating Treg lymphocytes [14]. A direct correlation was observed between the media to lumen ratio of subcutaneous small arteries and circulating Th17 lymphocytes [14], suggesting that some lymphocyte subpopulations may be related to microvascular remodelling, confirming previous animal data [14]. Significant relationships were observed between different subpopulations of circulating CD4+ T lymphocytes and microvascular or systemic oxidative stress in humans [15]; these data suggest that Treg lymphocytes may be protective against microvascular damage, probably because of their anti-oxidant properties, while Th1-Th17 lymphocytes seem to exert an opposite effect, confirming an involvement of adaptive immune system in microvascular damage [14,15]. Therefore, at least for hypertension, it seems that the evaluation of circulating immune cells might represent a new clinical target [16,17]. However, few data are available concerning adaptive immunity and obesity.

It was postulated that the adipocyte-derived hormone leptin, and thereby the nutritional status, could control immune self-tolerance by affecting Treg cell responsiveness and function [18]. Furthermore, resident Treg cells, which are capable of modulating metabolism and glucose homeostasis, are abundant in adipose tissue [18]. Oxidative stress may be an important link between linking immune response, metabolic stress and obesity [4]. A decreased Treg function was observed in rats with high-fructose diet-induced metabolic syndrome [19]. However, surprisingly, in mice a depletion of fat-resident Treg cells prevented age-associated insulin resistance [20]. However, in general, fat-resident Tregs are considered an emerging guard, protecting from obesity-associated metabolic disorders [21]. In fact, a reduction of circulating activated Treg cells, and an increase in OX40-expressing Treg cells in vascular adipose tissue were selectively observed in obese patients, and directly correlated with body mass index [22]. OX40 is a marker associated with a higher proliferative potential and suppressive activity [22]. Similarly, a lower omental Treg cell count is associated with higher fasting glucose and lower β-cell function in adults with obesity [23]. However, no data concerning circulating T-lymphocyte subtypes are presently available in patients with severe obesity. As mentioned, pronounced microvascular alterations were previously observed in patients with severe obesity; since lymphocytes subpopulations seems to be implicated in microvascular remodeling in hypertension [14], it is possible that this may be true also in obesity [7]. Therefore, we considered worthwhile to investigate different subpopulations of circulating CD4+ lymphocytes in severely obese patients undergoing bariatric surgery (with or without concomitant hypertension), as well as in normotensive lean controls and in hypertensive lean patients.

## Patients and methods

We have investigated thirty-two patients with abdominal severe obesity, defined according to current Italian guidelines [24], and admitted to the Surgical Department of our hospital for indication to bariatric surgery, in particular to a jejuno-ileal bypass or a biliopancreatic derivation.

The protocol of the study was approved by the Ethics committee of our institution (Medical School, University of Brescia), and a written informed consent was obtained from each participant. The procedures followed were in accordance with the institutional guidelines.

Eighteen obese patients were normotensive and 14 hypertensive, according to the European Society of Hypertension and of the European Society of Cardiology guidelines 2013 [25]. Controls were represented by 24 lean normotensive subjects and 12 lean patients with essential hypertension, undergoing standard surgery. The majority of the 14 hypertensive obese patients and of the 12 hypertensive lean patients were previously treated with various antihypertensive drugs, although there was no difference between groups in the proportion of treated patients as well as in the types of antihypertensive drugs. There was not a pre-specified matching of cases and controls, and the whole population investigated is representative of the population of patients usually admitted to our Surgical Department. The 36 lean controls were therefore enrolled consecutively and then, *post hoc*, subdived in hypertensives and normotensives.

Venous blood samples were taken for standard haematology and serum biochemistry tests (including triglycerides and total cholesterol) in obese patients and in lean subjects and patients.

### Evaluation of circulating CD4+ T lymphocyte phenotype

Peripheral blood CD4+ T-cell phenotypic characterization was performed by flow cytometry (Cytomics NAVIOS, Beckman Coulter Inc., Fullerton, CA), as previously reported [14,26]: 100 microliters of whole blood were incubated mixture of monoclonal antibodies (from Beckman Coulter, R&D Systems, Caltag Laboratories, Becton Dickinson, San Jose, CA), conjugated with fluorochromes at 4°C for 30 minutes. Double-negative CD3+CD4-CD8- T-cells were evaluated in all patients and no difference between normotensive and hypertensive individuals was observed. Absolute cell count was determined by single-platform analysis using Flow-Count beads (Beckman Coulter).

CD4+ Tregs were defined by the high expression of CD25 and the low expression/absence of CD127, using PC5-conjugated anti-CD4, PE-conjugated anti-CD25 and PC-7 conjugated anti-CD127 [27].

In addition, different subpopulations of Tregs were identified using, in two separate tubes, FITC-conjugated CCR7 or FITC-conjugated CD31 and ECD-conjugated CD45RA.

Subsets of Tregs were defined as follows: -Recent thymic emigrants (RTE), directly derived from thymus: CD31+; -Naïve: CCR7+CD45RA+; -Central memory (CM): CCR7+CD45RA-; -Effector memory (EM): CCR7-CD45RA-; -Terminal differentiated effector memory (TDEM): CCR7-CD45RA+. Finally, the number of EM was evaluated among all CD4+ T cells (CD4+EM).

**Fig 1:**
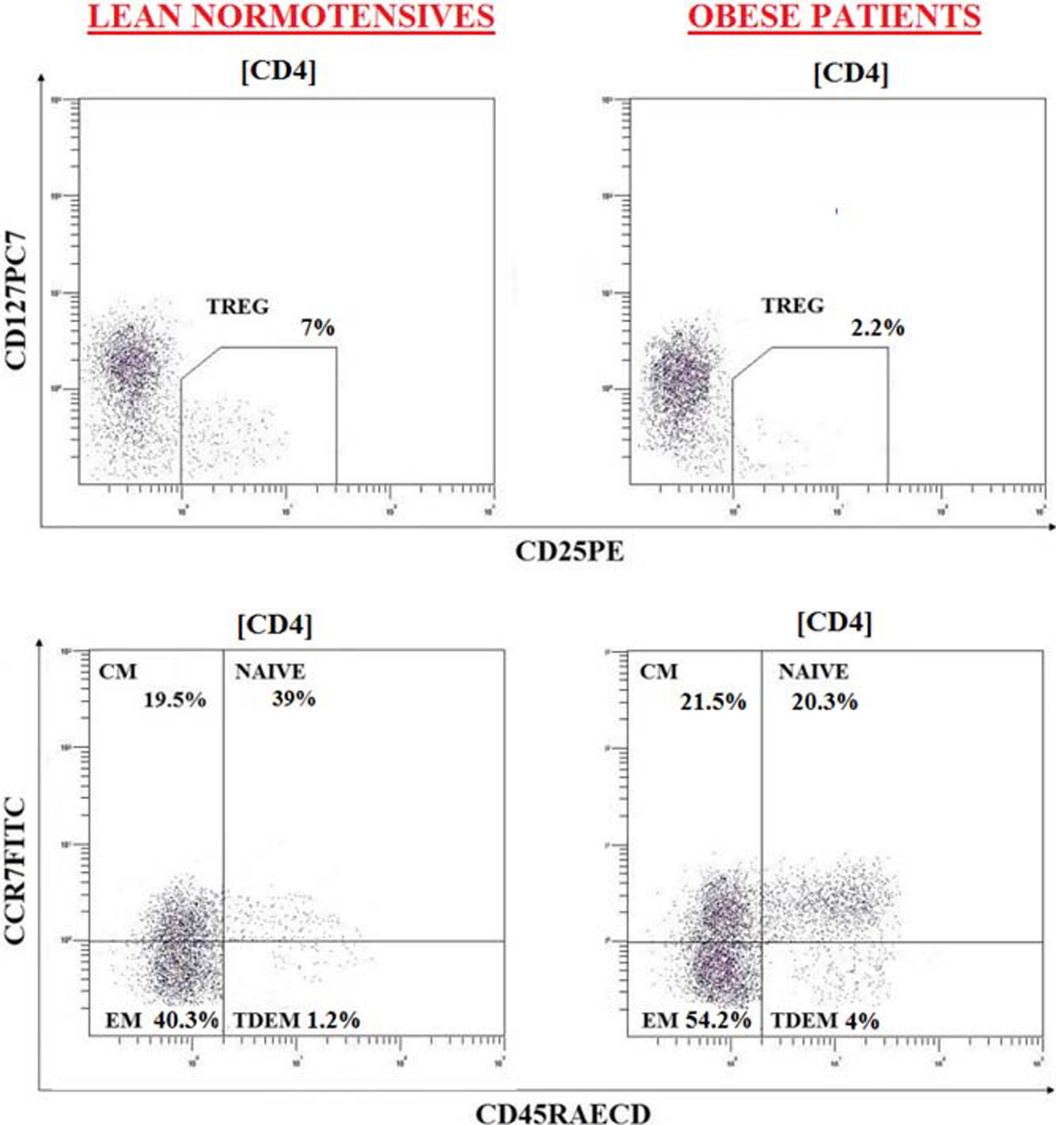
Flow cytometry evaluation (dot blot) in representative subjects of two subgroups (LEAN NORMOTENSIVES AND OBESE PATIENTS). Dot plot analysis is of CD4+ gated lymphocytes. At least 20.000 events were considered.

Examples of flow cytometry data and the gating strategy used are reported in Fig 1.

### Statistical analysis

All data are expressed as mean+SD, unless otherwise stated. Differences between groups were evaluated by one-way analysis of variance (ANOVA). Distribution of categorical variables between groups was evaluated by χ-square test. Relationships between variables were assessed by calculation of Pearson’s correlation. All parameters were normally distributed. All analyses were carried out with SPSS software (version 13.0, SPSS Inc., Chicago, Illinois, USA).

## Results

### Demographic data

Some demographic, hemodynamic and humoral data are reported in Table 1.

**Table 1:** Demographic and clinical characteristics of the population evaluated.

	Lean normotensive subjects (n = 24)	Lean hypertensive patients (n = 12)	Obese patients (n = 32)
**Gender (males: M)**	14M (58%)	7 M (58%)	7 M (28%)
**Age (years)**	48.1±15.0	60.9±8.83 *	42.0±14.5 ##
**Body mass index (Kg/m**^**2**^**)**	25.1±2.92	26.2±3.84	42.9±5.07 ***###
**Systolic blood pressure (mm Hg)**	122±10.9	134±17.0 *	129±18.1
**Diastolic blood pressure (mm Hg)**	76.3±9.35	76.6±9.96	82.0±12.6
**C reactive protein (mg/L)**	7.13±7.31	1.55±1.12	8.41±4.71 #
**Serum glucose (mg/dl**	90.7±11.9	98.4±15.3	103±37.5
**Serum creatinine (mg/dl)**	0.81±0.23	0.95 ±0.23	0.72±0.11
**Creatinine clearance (ml/min)**	94.9±23.6	76.9 ±16.5	116±34.8
**Blood urinary nitrogen (mg/ml)**	37.6±14.0	41.6±13.6	30.1±8.53
**Serum uric acid (mg/dl)**	4.52±1.42	5.54±1.84	4.68±1.28
**Serum sodium (mEq/L)**	141±2.00	142±1.53	140±2.06
**Serum potassium (mEq/L)**	3.87±0.47	3.93±0.47	3.80±0.28
**Serum chloride (mEq/L)**	105±2.43	105±2.31	105±1.74
**Serum triglycerides (mg/dl)**	76.7±33.9	93.2±30.8	119±57.7
**Serum cholesterol (mg/dl)**	179±25.2	180±34.2	185±28.8

* P<0.05,

*** P<0.001 vs. Lean normotensive subjects;

# P<0.05,

## P<0.001,

### P<0.001 vs. Lean hypertensive patients. Creatinine clearance: MDRD formula. Data are expressed as mean±standard deviation.

Lean hypertensive patients were significantly older compared with the other two groups. Systolic blood pressure was significantly higher in hypertensive lean patients compared with normotensive lean subjects. Fasting glucose was not different between groups although slightly greater in obese patients. Body mass index was significantly higher in obese patients compared with lean patients and subjects, as expected. Serum cholesterol or triglycerides were not significantly different among the groups. Serum creatinine values tended to be even lower in obese patients, while creatinine clearance calculated with the MDRD formula was slightly, not significantly lower in lean hypertensives compared with lean normotensives. Only three out of 32 obese patients had an overt diabetes mellitus.

### Evaluation of circulating CD4+ T lymphocytes

A marked reduction of several Treg subpopulations was observed in obese patients compared with controls, together with an increase in CD4+ EM T-effector cells (Table 2).

**Table 2:** Lymphocyte subpopulations in the different groups.

	Lean normotensives n = 24	Lean hypertensives n = 12	Obese patients n = 32	Obese normotensive patients (n = 18)	Obese hypertensive patients (n = 14)
Tregs (% CD4+)	4.11±1.60	4.64±1.66	2.69±1.81 **##	2.73±1.51 **##	2.64±2.19 *#
**Tregs (cells/μL)**	45.4±24.3	45.4±23.8	27.3±21.1 **#	30.4±20.7 *#	23.2±21.8 *#
**Tregs na**ï**ve (% Tregs)**	22.1±10.1	18.1±13.1	13.3±12.9 **	12.4±9.64 **	14.7±16.8
**Tregs na**ï**ve (cells/μL)**	10.6±7.75	9.71±8.87	3.87±5.28 ***##	4.57±6.16 *#	2.96±3.90 **##
**Tregs CM (% Tregs)**	32.3±13.8	32.8±17.8	22.7±15.2 *#	18.8±9.25 ***##	28.1±20.1
**Tregs CM (cells/μL)**	14.7±10.2	14.2±9.08	6.10±8.08 ***##	5.94±6.18 **##	6.31±10.3 *#
**CD4+ EM (% CD4+)**	24.4±9.96	26.8±12.5	34.1±13.3 **	35.0±12.9 **	32.8±14.3 *

Tregs = regulatory T cells; CM = central memory; EM = effector memory.

*p<0.05,

**p<0.01,

***p<0.001 vs. lean normotensives;

#p<0.05,

##p<0.01 vs. lean hypertensives. P = NS between obese normotensive and hypertensive patients. Data are expressed as mean±standard deviation.

No difference was observed between obese hypertensive patients and obese normotensive patients (Table 2).

**Fig 2:**
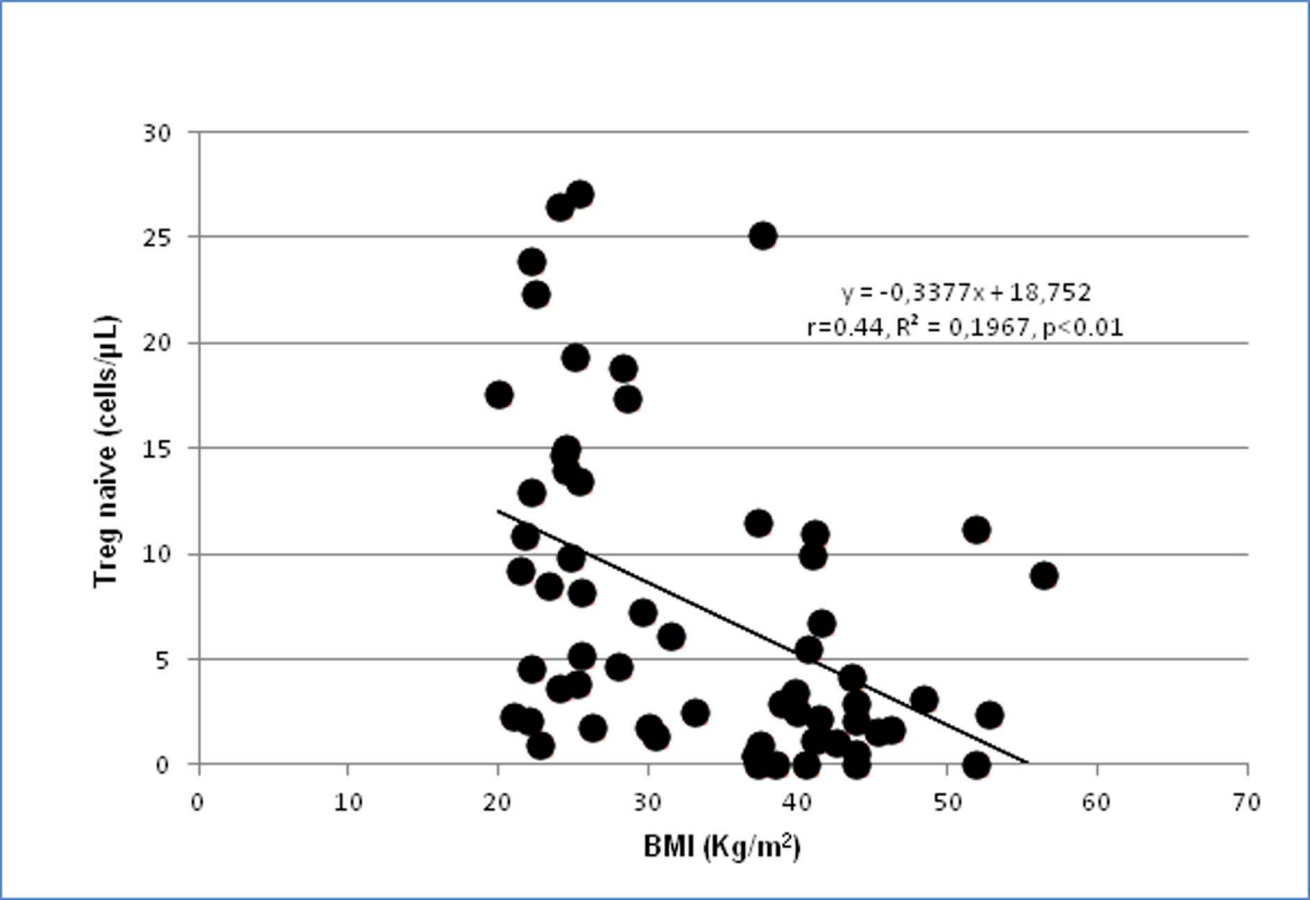
Correlation between body mass index (BMI) and circulating Treg naive (cell/μL) in the whole population of 68 subjects and patients.

**Fig 3:**
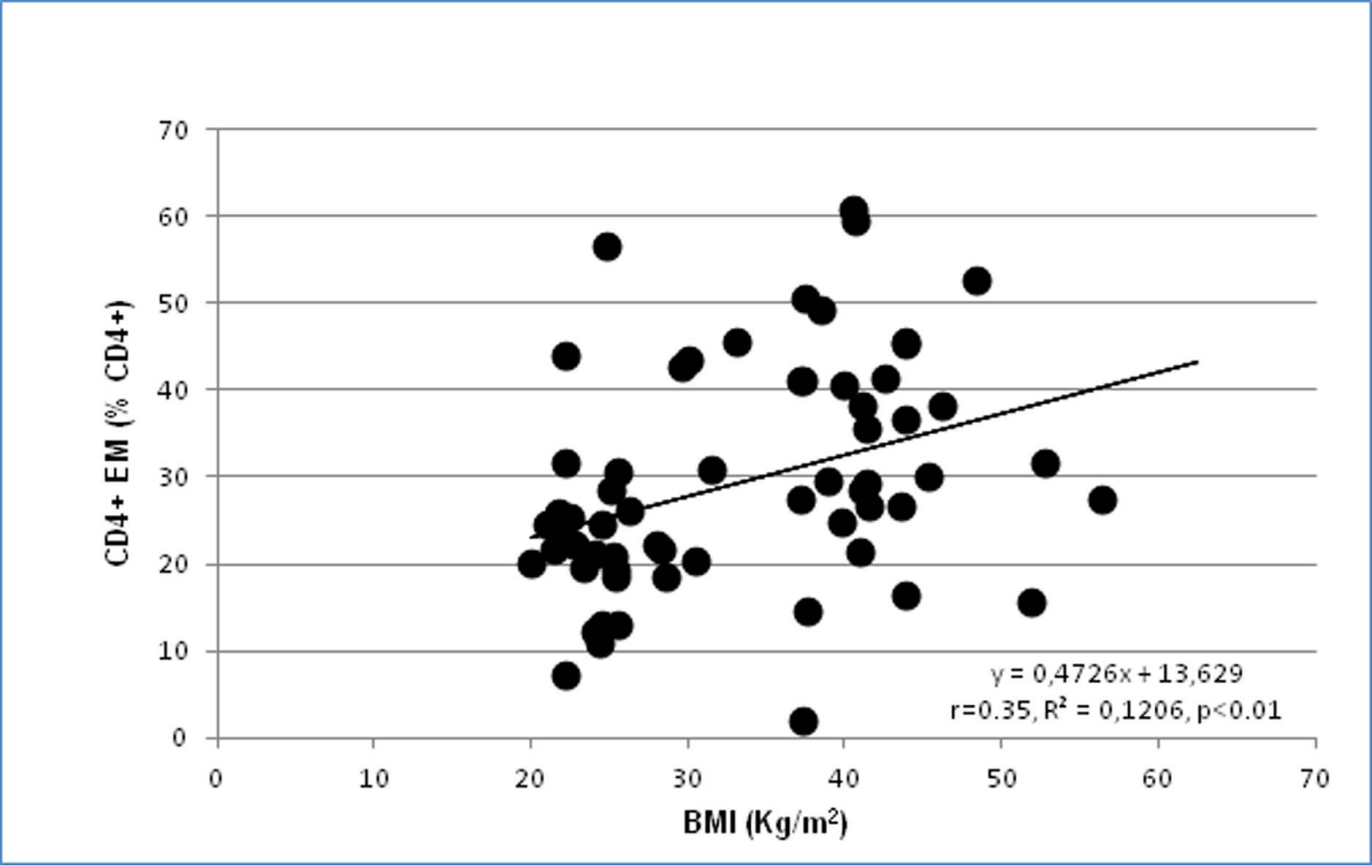
Correlation between body mass index (BMI) and circulating CD4+ effector memory (EM) cells (% CD4+) in the whole population of 68 subjects and patients.

No significant correlation between body mass index and lymphocytes subpopulations was observed in any group of subjects and patients, both considering the four groups separately and considering only the obese patients (n = 32). This might be due to the relative low number of patients included in the subgroups. In fact, when the whole group of 68 subjects and patients was considered, statistically significant inverse correlations between body mass index and Treg (%): r = -0.38, p<0.01; Tregs (cell/μL): r = -0.32,p<0.01; Tregs naïve (% Tregs): r = -030, p<0.05; Tregs naïve (cells/μL): r = -0.44, p<0.01 (Fig 2); Tregs CM (% Tregs): r = -025, p<0.05; Tregs CM (cells/μL): r = -0.41, p<0.001, while a direct correlation was observed with CD4+ EM (% CD4+): r = 0–35, p<0.01 (Fig 3).

Additional correlations observed between lymphocyte subpopulations and metabolic parameters are reported in Table 3.

**Table 3:** Statistically significant correlations between lymphocyte subpopulations and metabolic parameters.

Population	1st variable	2^nd^ variable	r	p
All subjects and patients, n = 68	CD4+ EM (% CD4+)	Serum cholesterol	0.24	P<0.05
All subjects and patients, n = 68	CD4+ EM (% CD4+)	Serum glucose	0.25	P<0.05
Lean normotensives n = 24	CD4+ EM (% CD4+)	Serum glucose	0.40	P<0.05
Lean hypertensives n = 12	CD4+ EM (% CD4+)	Serum cholesterol	0.62	P<0.05
Obese normotensive patients, n = 18	CD4+ EM (% CD4+)	Serum triglycerides	0.69	P<0.001
All subjects and patients, n = 68	Tregs (%)	Serum triglycerides	-0.27	P<0.05
All subjects and patients, n = 68	Tregs CM (% Tregs)	Serum triglycerides	-0.23	P<0.05
Lean hypertensives n = 24	Tregs (%)	Serum cholesterol	-0.57	P<0.05
Lean hypertensives n = 24	Tregs naïve (% Tregs)	Serum cholesterol	-0.81	P<0.001
Lean hypertensives n = 24	Tregs naïve (cells/μL)	Serum cholesterol	-0.70	P<0.01
Obese normotensive patients, n = 18	Tregs naïve (cells/μL)	Serum triglycerides	-0.56	P<0.01

## Discussion

In the present study for the first time a reduction in circulating Treg lymphocytes was observed in severely obese patients. In addition, an increase in circulating CD4+ effector memory cells was detected. Treg represent a small subset of CD4+T lymphocytes subset that can control the inappropriate adaptive immune responses by producing IL-10 and TGF-β, which are crucial for the balance and homeostasis of the immune system. Treg has been demonstrated to diminish in visceral adipose tissue from obese mice compared to lean animals, suggesting a possible role of these cells in modulating obesity-associated fat tissue inflammation [21].

Previous data in humans regarding Tregs are controversial, since both an increase and a reduction of Treg lymphocytes in fat tissues was observed [22,23]. In fact, a selelective increase in visceral adipose tissue of a subset of Treg cell population expressing high levels of OX40, a marker associated with Treg survival and suppressive, was observed in obese individuals, which directly correlated with body mass index [22]. On the contrary, another study reported a lower omental Treg cell count in adults with obesity which was associated with higher fasting glucose and lower β-cell function [23]. Circulating Treg cells were also demonstrated to be reduced in obese individuals and their number inversely correlated with body mass index [22], biomarkers of inflammation, weight, and leptin levels [28].

It has been previously demonstrated that Treg lymphocytes may exert a protective role in terms of development/regression of microvascular alterations in human hypertension [14]. Alteration in microvascular structure similar to those observed in hypertension has been recently observed also in obese patients [7]. Severe obese normotensive subjects show hypertrophic remodelling of the subcutaneous small resistance arteries wih an increased vascular wall thickness and media-to-lumen ratio, as well as an endothelial dysfunction [7, 29,30].

Since hypertension and obesity share some common mechanisms, including an activation of the sympathetic nervous system and of the renin-angiotensin system, insulin resistance, increased leptin levels, systemic inflammation, endothelial dysfunction and oxidative stress [30,31]; it is therefore possible that also in obesity the adaptive immune system might play a relevant role [4,18] and that inflammation/oxidative stress might represent a possible link [32].

Indeed, experimental models of metabolic syndrome, such as rats with high-fructose diet and the New Zealand Obese mice, show an increases in vascular and perivascular fat oxidative stress [19]. In addition, a decreased Treg function was observed in rats with high-fructose diet [19] resulting in reduced IL-10 in vitro secretion compared with controls. Interestingly, mice deficient in fat-resident Treg lymphocytes are susceptible to obesity-associated insulin resistance and metabolic disease [20]; viceversa, adipose tissue Treg deficiency protects against age-associated insulin resistance suggesting different underlying mechanisms of the two conditions. These data suggest that fat-resident Tregs, which are aboundantly present in normal adipose tissue, seem to elicit a protecting role in obesity [21] by modulating metabolism and glucose homeostasis. Indeed, a reduction of Treg percentage in peripheral blood of children with metabolic syndrome was observed [19]. Moreover, it was suggested that the increased serum leptin levels, observed in obesity, could control immunity by negatively affecting Treg cell proliferative capacity and function. Accordingly, leptin and leptin receptor deficiencies are characterized by increased Treg number and activitiy in experimental model; similarly, Treg proliferative capacity is inversely related with the serum leptin levels in humans. In addition, leptin polarizes T helper cytokine production towards a proinflammatory (Th1, gamma-interferon, tumoral necrois factor- alpha) rather than anti-inflammatory phenotype (Th2, interlleukin-4) then further amplifying the network of inflammatory signaling pathways [18].

Indeed, an increase in circulating CD4+ effector memory cells was observed in our study. CD4+ effector memory cells represent one of the populations of lymphocytes more involved in the production of pro-inflammatory cytokines [33,34]. In fact, upon antigen re-exposure, CD4+ effector memory cells immediately migrate from secondary lymphoid organs to inflamed tissue, where they exert effector functions [35]. In agreement with our results, many studies showed a positive correlation between CD4+ effector memory cells and pro-inflammatory conditions, such as aging and atherosclerosis, confirming that obesity and inflammation are strictly related [6,36,37].

Moreover, it is generally accepted that perivascular fat might possess anticontractile and vasculoprotective properties, partially mediated by adiponectin incretion [38]. Such protective properties are lost in human obesity due to local inflammation and hypoxia [38,39]. Also infiltrating immune cells may contribute to adipose tissue dysfunction [40].

Increases in vascular and perivascular fat oxidative stress and inflammation in the metabolic syndrome could contribute to the development of cardiovascular disease. As mentioned, the presence of obesity [7], with or without other components of the metabolic syndrome [29] seems to be associated with hypertrophic remodeling of subcutaneous small resistance arteries. Hypertrophic remodelling (vascular smooth muscle cells hypertrophy or hyperplasia), such as that observed in diabetic or obese patients, seems to be associated with an even worse prognosis, compared with eutrophic remodeling (re-arrangement of the same vascular wall material around a narrowed lumen) [41]. Therefore, the observation of a decreased circulating number of a protective subset of lymphocytes, as well as of an increase in CD4+ effector memory cells might open clinical and therapeutic perspectives also in obesity. As mentioned, subsets of T lymphocytes have been implicated in the pathogenesis of hypertension and vascular remodeling [14,16,42,43]. This is an area of active research and rapid development in cardiovascular and renal disease [42,43], and the possibility to modulate circulating and vascular/tissue lymphocytes by appropriate treatment is relatively close, in particular using monoclonal antibodies or immunosuppressant drugs in order to inhibit the effects of pro-inflammatory lymphocytes [14,42,44,45].

In fact, novel data, mostly coming from experimental and preclinical studies, identify new potential targets related with the immune system, which may open therapeutic options [16]. As mentioned, few but important emerging findings are now indicating the possible contribution of innate and adaptive immune cells in the cardiovascular damage also in humans [46], and the present study contributes in this regard. Thus, interventions aimed at reducing proinflammatory T-effector lymphocytes and stimulating immunosuppressive T regulatory activity could be possibly useful in order to control blood pressure and prevent/limit target-organ damage [16] and modulate the inflammatory conditions and immune dysfunction associated with obesity.

### Limitations of the study

We did not measure circulating indices of oxidative stress/inflammation in our population. In addition, we did not investigate Treg in the fat tissues. Therefore we cannot provide information about mechanisms involved in the observed difference in Treg lymphocytes, as well as about pathophysiological consequences. Similarly, we have no data about the effects of weight loss on circulating lymphocyte subpopulations. In our study many groups and variables were compared, which increases the likelihood of significant results. In general, the relatively low number of subjects and patients enrolled limits the power of the study.

In conclusion, our data suggest that, in severely obese patients, Treg lymphocytes are clearly reduced and CD4+ effector memory cells are increased. It cannot be excluded that they may contribute to the development of marked microvascular alterations previously observed in these patients.
